# Systematic Assessment of Human CCR7 Signalling Using NanoBRET Biosensors Points towards the Importance of the Cellular Context

**DOI:** 10.3390/bios14030142

**Published:** 2024-03-14

**Authors:** Nathan Vanalken, Katrijn Boon, Martyna Szpakowska, Andy Chevigné, Dominique Schols, Tom Van Loy

**Affiliations:** 1 KU Leuven, Department of Microbiology, Immunology and Transplantation, Rega Institute for Medical Research, Laboratory of Virology and Chemotherapy, 3000 Leuven, Belgium; nathan.vanalken@kuleuven.be (N.V.); katrijn.boon@kuleuven.be (K.B.); dominique.schols@kuleuven.be (D.S.); 2 Department of Infection and Immunity, Immuno-Pharmacology and Interactomics, Luxembourg Institute of Health, L-4354 Esch-sur-Alzette, Luxembourg; martyna.szpakowska@lih.lu (M.S.); andy.chevigne@lih.lu (A.C.)

**Keywords:** G protein-coupled receptor, chemokine receptor, CCR7, ligand bias, biased signalling, G protein, β-arrestin, NanoBRET

## Abstract

The human CC chemokine receptor 7 (CCR7) is activated by two natural ligands, CC chemokine ligand 19 (CCL19) and 21 (CCL21). The CCL19-CCL21-CCR7 axis has been extensively studied in vitro, but there is still debate over whether CCL21 is an overall weaker agonist or if the axis displays biased signalling. In this study, we performed a systematic analysis at the transducer level using NanoBRET-based methodologies in three commonly used cellular backgrounds to evaluate pathway and ligand preferences, as well as ligand bias and the influence of the cellular system thereon. We found that both CCL19 and CCL21 activated all cognate G proteins and some non-cognate couplings in a cell-type-dependent manner. Both ligands recruited β-arrestin1 and 2, but the potency was strongly dependent on the cellular system. Overall, CCL19 and CCL21 showed largely conserved pathway preferences, but small differences were detected. However, these differences only consolidated in a weak ligand bias. Together, these data suggest that CCL19 and CCL21 share mostly overlapping, weakly biased, transducer profiles, which can be influenced by the cellular context.

## 1. Introduction

Chemokines are a group of small proteins that orchestrate a wide array of physiological functions through intricate signalling networks. Most notably, they play a pivotal role in governing the precise migration of immune cells and regulating their spatial and temporal positioning [1,2,3]. Chemokines function through interaction with their respective receptors, which belong to the family of G protein-coupled receptors (GPCRs) [1,2,4]. Like other GPCRs, chemokine receptors, with exception of the atypical chemokine receptors [5], signal through the activation of heterotrimeric G proteins (Gαβγ). These G proteins are classified in four subfamilies (Gαi/o, Gαs, Gαq/15 and Gα12/13). Chemokine binding induces conformational changes in the receptor protein, which results in the activation (dissociation) of the G protein into Gα and Gβγ subunits that further mediate downstream signalling. To limit further signalling, the receptor intracellular loops or C-terminus are phosphorylated, primarily by G protein-coupled receptor kinases (GRKs), facilitating β-arrestin binding and subsequent receptor desensitisation and internalisation [6].

In humans, over 40 chemokines and 20 chemokine receptors have been described to date. Together, they form a network in which many receptors interact with multiple chemokines. Also, some chemokines can stimulate more than one receptor. In recent years, emerging evidence has indicated that this promiscuity within the chemokine system does not simply reflect redundancy, but might allow for more nuanced regulation of chemokine signalling. Biased signalling or functional selectivity is one potential mechanism that can contribute to the specific control of the chemokine signalling network [7,8,9,10,11,12]. Biased signalling can be mediated through each component of the ternary GPCR complex. Different ligands that interact with the same receptor, but stabilise different receptor conformations, can induce distinct downstream signalling pathways (ligand bias). A single ligand interacting with multiple receptors can elicit different receptor-dependent pathways (receptor bias). Additionally, depending on the cellular context, transducers (e.g., G proteins, GRKs, etc.) can be differentially expressed thereby skewing the signalling pathways induced by a particular ligand–receptor interaction (system bias) [11]. In the context of GPCR activation, a classic example of ligand bias is when ligands preferentially induce G protein activation over β-arrestin recruitment [13]. GPCRs can also couple to different G proteins; therefore, bias agonism should also be investigated between G protein subtypes, as they can mediate different signalling outcomes [14,15].

A prototypical example of biased signalling is the human CC chemokine receptor type 7 (CCR7). CCR7, together with its two CC chemokine ligands 19 (CCL19) and 21 (CCL21), is an important part of the chemokine signalling system. CCR7 is predominantly expressed in various T cell populations and mature dendritic cells (mDC). CCR7 and its ligands regulate the homing of immune cells to secondary lymphoid organs and define the position of these cells within the organ architecture [16,17,18]. As such, the CCR7-CCL19/CCL21 axis serves as a key regulator for both immunity and tolerance. Even though CCL19 and CCL21 stimulate the same receptor, they only share 32% sequence identity. Also, CCL21 harbours a unique positively charged 32-amino acid long C-terminal tail that is important for binding to glycosaminoglycans (GAGs) and other molecules [19]. Additionally, CCL21 signalling can be influenced by the polysialylation of CCR7 [20].

CCR7 signalling has been investigated extensively in vitro to better understand and document the potential ligand bias between CCL19 and CCL21 upon interaction with CCR7. Studies show that CCL19, compared to CCL21, more potently recruits β-arrestins and induces more pronounced receptor internalisation. However, regarding G protein activation, calcium release and chemotaxis reports are equivocal. Hence, whereas some studies consider the CCL19-CCL21-CCR7 axis to be a prototypical example of biased signalling, others challenge this notion and propose that CCL21 is an overall weaker agonist [21,22,23,24,25,26,27,28,29]. Given that a genuine comparison across studies is difficult due to the use of different cellular backgrounds and methodologies, we performed a systematic analysis of the CCR7 signalling at the transducer level in three commonly used cellular backgrounds using a NanoBRET-based methodology and precisely quantified pathway and ligand preferences, as well as ligand bias and the influence of the cellular system thereon.

## 2. Materials and Methods

### 2.1. Cell Lines, Plasmids and Reagents

HEK293A cells were kindly provided by Dr. A. Inoue (Tohoku University, Sendai, Japan). U87, formerly known as U87.MG, cells and CHO-K1 cells were purchased from ATCC (Manassas, VA, USA). hCCR7 (#CCR0700000) in a pcDNA3.1(+) vector was purchased from the cDNA Resource Centre. The REGA-SIGN plasmids [30] and hCCR7-mNeongreen, NLuc-Arrβ1(N) and NLuc-Arrβ2 [31] were previously described. The pcDNA3.1(+) hCCR7 plasmid was used to generate HEK293A, U87 and CHO-K1 cells stably expressing hCCR7 with similar expression levels (Supplementary Figure S1). CCR7 expression at the cell surface was confirmed via flow cytometry using PE Mouse anti-Human CCR7 (Clone 150503, BD Pharmingen, Franklin Lakes, NJ, USA) and PE Mouse IgG2a κ Isotype Control (Clone G155-178, BD Pharmingen, Franklin Lakes, NJ, USA). HEK293A and U87 cells were grown in Dulbecco’s Modified Eagle Medium, high glucose (DMEM; #41965, Thermo Fisher Scientific, Waltham, MA, USA) containing 10% Fetal Bovine Serum (FBS; #10270106, Thermo Fisher Scientific, Waltham, MA, USA). CHO-K1 cells were cultured in Ham’s F-12 Nutrient Mix (#21127022, Thermo Fisher Scientific, Waltham, MA, USA) supplemented with 10% FBS. To select hCCR7-expressing cells, all cell culture media were supplemented with 500 μg/mL Geneticin (#10131, Thermo Fisher Scientific, Waltham, MA, USA). Recombinant CCL19 (#300-29B) and CCL21 (#300-35A) were ordered from PeproTech (Cheshire, UK).

### 2.2. G Protein Activation Assay

A recently described NanoBRET-based G protein activation assay [30] was used to study CCR7-mediated G protein activation. In brief, HEK293A, U87 or CHO-K1 cells stably expressing hCCR7 were transiently co-transfected in suspension with NanoLuc (NLuc)-tagged Gα protein of interest (donor) and their respective Gγ protein tagged with LSS-mKate2 (acceptor). The Gα and Gγ protein plasmids were transfected at a 1:10 donor-acceptor ratio using a 3:1 FuGENE^®^ HD Transfection Reagent-to-DNA ratio (#E2311, Promega, Madison, WI, USA). The final acceptor plasmid concentration was 1 µg of plasmid DNA per mL. The FuGENE^®^ HD Transfection Reagent/DNA mixture containing 10 ng/µL of DNA was left to incubate (10 min, room temperature) and subsequently added to the cell suspension. Transfected cells were immediately dispensed (30,000 cells/well) in white 96-well plates with a clear flat bottom that was pre-coated with 100 µg/mL of poly-D-lysine. After a 48 h incubation at 37 °C and 5% CO_2_, cells were washed with assay buffer (Hank’s Balanced Salt Solution (HBSS; #14065, Thermo Fisher Scientific, Waltham, MA, USA) and 20 mM of HEPES buffer (#15630-080, Thermo Fisher Scientific, Waltham, MA, USA), 0.5% FBS, pH 7.4). Cells were then incubated with 90 µL/well of a 1:100 Nano-Glo^®^ Vivazine™ working solution (#N2581, Promega, Madison, WI, USA) for 45 min at 37 °C and 5% CO_2_. The cell plate was transferred to the FLIPR Penta (Molecular Devices, San José, CA, USA) and left to stabilise for 15 min. Baseline BRET values were measured every 2.5 s for a total of 15 s. Thereafter, 10 µL/well of 10X ligand was added automatically to the plate by the FLIPR Penta, and BRET values were monitored in real time every 2.5 s for a total of 25 min. BRET ratios were measured using a 440–480 nm donor emission filter (#0200-6179, Molecular Devices, San José, CA, USA) and a 615 nm AT600lp acceptor emission filter (#296420, Chroma, Windham County, VT, USA).

### 2.3. β-Arrestin Recruitment Assay

β-arrestin recruitment was monitored with a NanoBRET assay [31]. Wild-type HEK293A, U87 and CHO-K1 cells were transiently co-transfected with CCR7 C-terminally tagged with mNeonGreen and either NLuc-Arrβ1(N) or NLuc-Arrβ2(N). Cell transfection and assay was performed using the method described above. Measurements in the FLIPR Penta were conducted using a 440–480 nm donor emission filter (#0200-6179, Molecular Devices, San José, CA, USA) and a 515–575 nm acceptor emission filter (#0200-6203, Molecular Devices, San José, CA, USA).

### 2.4. Data Analysis

Raw relative light units (RLU) were used to calculate BRET ratios by dividing the acceptor RLU by the donor RLU. BRET ratios were normalised to a baseline, which was defined as the mean BRET ratio of a 5-point run-in time prior to ligand addition. Normalisation was performed by dividing BRET ratios at all timepoints following ligand addition by the baseline. Technical replicates of these normalised readouts were then averaged and background-corrected by subtracting the values of their vehicle controls at each timepoint, resulting into a normalised background-corrected BRET value (NBC BRET). These kinetic responses were reduced to a single value by calculating the area-under-the-curve (AUC). Concentration–response curves were fitted to AUC values scaled to the maximal CCL19 AUC in R version 4.2.3 using the drm function (LL.4) from the drc (version 3.0) R package with the slope fixed to −1 (Equation (1)).

(1) $$f(x)=bottom+\frac{top-bottom}{1+exp\;(slope(\log\left(x\right)-\log\left(EC50\right))}$$

The EC50 expressed in Molar and E_max_ expressed as a fraction were used to calculate an approximated transducer coefficient, Log(E_max_/EC50), as described here [12]. Log(E_max_/EC50) was then used to calculate the pathway, ligand, and cell-type preferences. Pathway preference was calculated by subtracting the Log(E_max_/EC50) of a reference pathway from the Log(E_max_/EC50) of pathway of interest for a specific ligand (Equation (2)). Ligand preference was calculated by subtracting the Log(E_max_/EC50) of a reference ligand from the Log(E_max_/EC50) of a ligand of interest for a specific pathway (Equation (3)). From the ligand preference, we then calculated the ligand bias (Equation (4)) by subtracting the ΔLog(E_max_/EC50) of a reference pathway from the ΔLog(E_max_/EC50) of a pathway of interest. Cell-type preference was calculated by subtracting the Log(E_max_/EC50) of a reference cell type from the Log(E_max_/EC50) of a cell type of interest for a specific ligand and pathway (Equation (5)). Statistical analysis was performed in the manner indicated in the figure or table legends.

(2) $$∆{Log\left(\frac{E_{max}}{EC50}\right)}_{lig\;x}^{G\alpha x}={Log\left(\frac{E_{max}}{EC50}\right)}_{lig\;x}^{G\alpha x}-{Log\left(\frac{E_{max}}{EC50}\right)}_{lig\;x}^{G\alpha \;ref}$$

(3) $$∆{Log\left(\frac{E_{max}}{EC50}\right)}_{lig\;x}^{G\alpha x}={Log\left(\frac{E_{max}}{EC50}\right)}_{lig\;x}^{G\alpha x}-{Log\left(\frac{E_{max}}{EC50}\right)}_{lig\;ref}^{G\alpha x}$$

(4) $$∆∆{Log\left(\frac{E_{max}}{EC50}\right)}_{lig\;x}^{G\alpha x}={∆Log\left(\frac{E_{max}}{EC50}\right)}_{lig\;x}^{G\alpha x}-{∆Log\left(\frac{E_{max}}{EC50}\right)}_{lig\;x}^{G\alpha \;ref}$$

(5) $$∆{Log\left(\frac{E_{max}}{EC50}\right)}_{lig\;x}^{G\alpha x\;|\;Cell\;x}={Log\left(\frac{E_{max}}{EC50}\right)}_{lig\;x}^{G\alpha x\;|\;Cell\;x}-{Log\left(\frac{E_{max}}{EC50}\right)}_{lig\;x}^{G\alpha x\;|\;Cell\;ref}$$

## 3. Results

To systematically characterise the signalling profiles of the endogenous CCR7 ligands, CCL19 and CCL21, we looked at the dissociation of 11 G proteins (Gαi1, Gαi2, Gαi3, GαoA, GαoB, Gαq, Gα12, Gα13, Gα15, GαsS and GαsL) across the four G protein families (Gαi/o, Gαq/15, Gα12/13 and Gαs), as well as the recruitment of two β-arrestins (Arrβ1 and Arrβ2) in three cellular backgrounds (HEK293A, U87 and CHO-K1).

**Figure 1:**
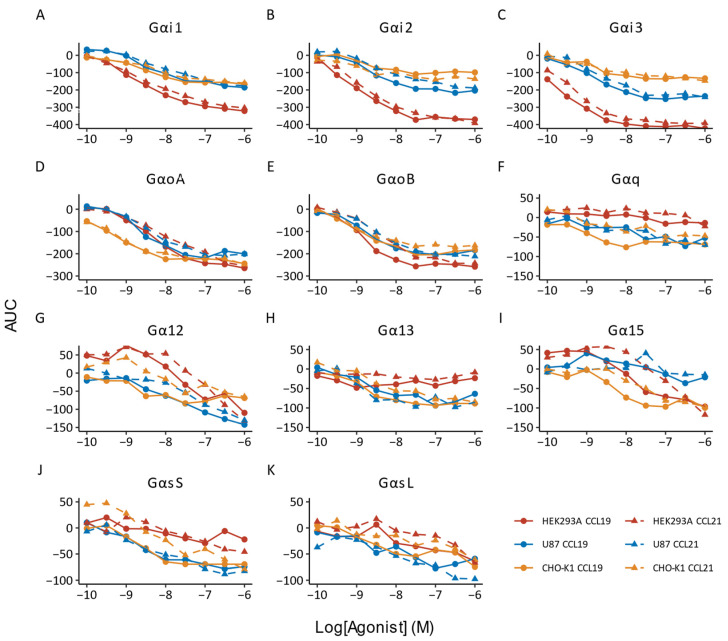
G protein activation by CCR7. (**A**–**K**) G protein dissociation in response to CCL19 (solid lines, circle) and CCL21 (dashed lines, triangle) was monitored for 11 Gα subunits in HEK293A (red), U87 (blue), or CHO-K1 (yellow) cells expressing CCR7. Data are represented as the mean of three to six independent experiments, each with three technical replicates. Error bars were omitted for clarity. Data for Gαi1, Gαi2 and Gαi3 activation in HEK293A cells were previously published in Vanalken et al. [24].

To monitor ligand-induced G protein activation, we used a suite of recently developed NanoBRET-based biosensors [30]. These biosensors measure the dissociation of 11 different NLuc-tagged Gα subunits (donor) and a Gγ subunit tagged with LSS-mKate2 (acceptor) following receptor activation. The unique combination of NLuc and LSS-mKate2, a red-shifted fluorophore for enhanced spectral separation, and the availability of a stable substrate allows for more sensitive and kinetic measurements, respectively [30]. We assessed which G proteins were activated by performing concentration–response experiments and performing a one-way ANOVA to assess whether ligand addition resulted in statistically significant changes (Figure 1).

In the HEK293A cellular context, stimulation with CCL19 and CCL21 induced significant concentration-dependent activation of the Gαi (Gαi1, Gαi2 and Gαi3), as previously published in Vanalken et al. [24], and Gαo families (GαoA and GαoB) (one-way ANOVA, *p* < 0.05). Additionally, both ligands induced significant activation of Gα12 and Gα15, but not Gαq and Gα13. We also observed activation of GαsL after stimulation with both ligands, but not GαsS, which was only activated following CCL21 stimulation. Similar results were obtained in U87 cells, where CCL19 and CCL21 induced the activation of the Gαi/o and Gαs families, as well as Gα12. In contrast to HEK239A cells, Gαq and Gα13, but not Gα15, were activated following receptor stimulation. In CHO-K1 cells, CCL19 and CCL21 elicited GαsL and Gα12 activation, respectively. All other G proteins showed significant concentration-dependent activation in response to both ligands. For the G proteins that were activated, we determined the efficacy and potency (Table 1; Supplementary Figure S2).

CCL19 and CCL21 displayed similar efficacy in HEK293A cells for the cognate Gαi/o family couplings. In contrast to the cognate couplings, Gα12 reached a significantly higher E_max_ when stimulated with CCL21. Except for GαoA, Gα12 and Gα15, where CCL19 was significantly more potent, potencies were markedly similar, with CCL19 being slightly more potent across the board. With regard to U87 cells, no major differences were detected in terms of efficacy and potency. This result was reflected in the CHO-K1 background, where efficacy, with the exception of Gαi1, and potency were similar for CCL19 and CCL21.

**Figure 2:**
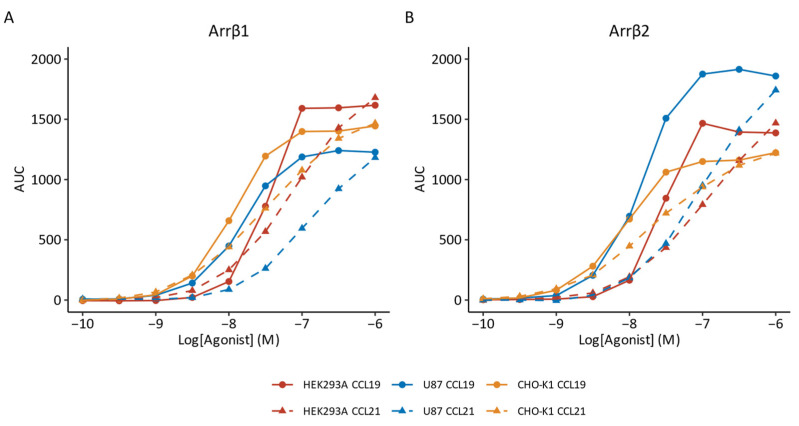
β-arrestin recruitment by CCR7. (**A**,**B**) β-arrestin recruitment in response to CCL19 (solid lines, circle) and CCL21 (dashed lines, triangle) was monitored for (**A**) β-arrestin1 (Arrβ1) or (**B**) β-arrestin2 (Arrβ2) in HEK293A (red), U87 (blue) or CHO-K1 (yellow) cells expressing CCR7. Data are represented as the mean of three to six independent experiments, each with three technical replicates. Error bars were omitted for clarity.

Next, we assessed ligand-induced β-arrestin recruitment to CCR7 using a NanoBRET-based biosensor [31] (Figure 2). The recruitment of β-arrestin to the receptor results in an increase in BRET signal. We found that both non-visual arrestins were recruited to CCR7 in a concentration-dependent manner following exposure to either CCL19 or CCL21, regardless of the cellular background. CCL19 and CCL21 displayed full agonistic properties reaching similar E_max_ values (Table 1; Supplementary Figure S2). In CHO-K1 cells, CCL21 was marginally less potent than CCL19. This difference was significantly more pronounced in U87 cells for both β-arrestin1 and β-arrestin2. In HEK293A cells, however, CCL19 was significantly more potent than CCL21 at inducing the recruitment of β-arrestin2, but not β-arrestin1.

**Figure 3:**
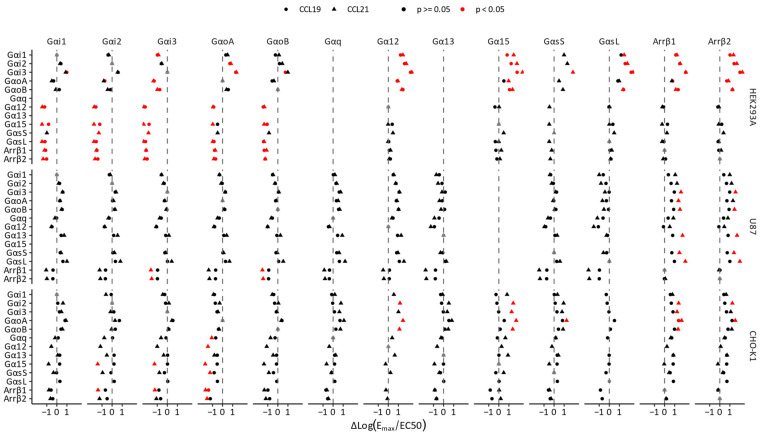
Pathway preference of CCL19 and CCL21. Pathway preferences (ΔLog(E_max_/EC50) were calculated for each ligand by subtracting the Log(E_max_/EC50) value of a reference transducer from all other transducers for each ligand (Equation (2)). Reference transducers are depicted at the top, with the transducer of interest on the left and the cell types on the right. Stronger differential activation of a transducer compared to the references results in a positive value, while weaker activation results in a negative value. If differences are significant, values are depicted in red. Data represent the mean of three to six independent experiments. One-way ANOVA followed by a Dunnett multiple comparison was used to assess the preference for the transducers over the reference. Black and red indicate *p* > 0.05 and *p* < 0.05, respectively.

Ligands can display differential activity across pathways, referred to as pathway preference. Here, we used Log(E_max_/EC50) instead of the operational model to investigate whether CCL19 or CCL21 preferentially activated certain transducers over others. We calculated the relative activity, ΔLog(E_max_/EC50), by subtracting the Log(E_max_/EC50) of a reference transducer from the other transducers for each ligand within a specific cellular system (Equation (2); Figure 3). Importantly, this is not a measurement of bias, as this requires normalisation to a reference ligand first. In HEK293A cells, CCL19 induced preferential activation of Gαi1 compared to Gα12, Gα15, GαsL, β-arrestin1 and β-arrestin2. Gαi3 seemed to be the most prominent CCR7 coupling in HEK293A cells, as it was slightly more engaged than Gαi2 and significantly more engaged than all the other transducers. Overall, in contrast to non-cognate couplings (Gα12, Gα15, GαsS and GαsL) and β-arrestins, cognate (Gαi1, Gαi2, Gαi3, GαoA and GαoB) couplings were more prominently activated following CCL19 stimulation. In U87 cells, CCL19 induced a more promiscuous G protein coupling pattern with similar relative activities. Instead of Gαi3, GαoA was the dominant coupling in CHO-K1 cells; however, the observed differences were not significant for CCL19. Furthermore, GαoA was preferentially activated compared to β-arrestin1. In general, CCR7 showed similar preferential activation trends in response to CCL19 and CCL21, although some substantial differences between the two ligands were detected.

To quantify the difference in relative activation induced by CCL19 and CCL21 for a specific transducer, we quantified the ligand preference (Supplementary Table S1). Here, the relative effectiveness, ΔLog(E_max_/EC50), was calculated by subtracting the Log(E_max_/EC50) of CCL19, serving as a reference, from CCL21 in each pathway (Equation (3)).

CCL21 induced Gα15 and β-arrestin2 activation less effectively than CCL19 in HEK293A. Similar to their potency differences, CCL19 more prominently induced β-arrestin1 and β-arrestin2 recruitment in U87 cells, which was in line with CCL21 preferentially activating Gαi3, GαoA and GαoB over β-arrestin1. In CHO-K1 cells, CCL19 and CCL21 displayed no differences.

**Figure 4:**
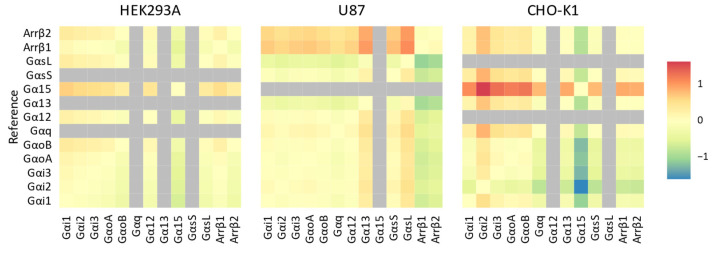
Ligand bias of CCL21 at the CCR7. Ligand bias (ΔΔLog(E_max_/EC50) was calculated for CCL21 with CCL19 as the reference ligand by first subtracting the log(E_max_/EC50) of CCL19 from the log(E_max_/EC50) of CCL21 (Equation (3)), followed by subtracting the Δlog(E_max_/EC50) of a reference transducer from the Δlog(E_max_/EC50) of all other transducers (Equation (4)). The ΔΔLog(E_max_/EC50) is depicted in a heatmap with the reference transducer on the left. The bias of a transducer away from the reference results in a positive value (red), while bias towards the reference results in a negative value (blue). Transducers in grey were not activated.

We wanted to establish whether the previous observations consolidated into a quantifiable ligand bias (Figure 4). The ligand bias was calculated by subtracting the ΔLog(E_max_/EC50), normalised to CCL19, of a reference pathway from the ΔLog(E_max_/EC50) of other pathways (Equation (4)). Using CCL19 as the reference ligand, we identified only weak, non-significant ligand bias between transducers within a unique cellular background (one-way ANOVA, *p* < 0.05). In U87 cells, CCL21 showed no bias towards any of the cognate G proteins in comparison to one another, but CCL21 was slightly biased towards most G proteins with respect to both β-arrestins. In CHO-K1 cells, CCL21 was slightly biased towards Gαi2 and away from Gα15 compared to all other transducers.

Lastly, we probed whether the cellular system influences the activation of specific transducers (Supplementary Table S2). Here, we calculated the relative activation, ΔLog(E_max_/EC50), by subtracting the Log(E_max_/EC50) of a reference cell type from each cell type in a pathway (Equation (5)). Our results indicated that Gαi3 was significantly less activated in U87 and CHO-K1 cells compared to HEK293A cells following CCL19 stimulation. β-arrestin2 was more readily recruited in CHO-K1 in response to both ligands compared to HEK293 cells. GαoA was the dominant coupling in CHO-K1 cells, which was reflected here with GαoA activation being significantly lower in HEK293A and U87 cells than in CHO-K1 cells.

## 4. Discussion

Activation of CCR7 by its ligands CCL19 and CCL21 is often referred to as a prototypical example of biased signalling. Early studies indeed suggested similarities in G protein activation by CCL19 and CCL21 but differences in GRK and β-arrestin recruitment, as well as receptor internalisation. Later studies challenged this notion, suggesting that CCL21 is a partial agonist with both impaired G protein activation and β-arrestin recruitment [21,22,23,24,25,26,27,28]. Many of these studies, however, used different cell lines and/or methodologies and do not quantify biased signalling in a similar way, if at all, hindering direct comparison between studies. Rather, they mostly rely on reporting differences in potencies and efficacies between assays to facilitate their conclusion. Here, we reported a systematic assessment of the transducer profile following CCR7 stimulation with CCL19 and CCL21 in several cellular backgrounds with a single methodological approach to provide a consistent quantification of ligand bias. In particular, we used NanoBRET-based biosensors to directly probe G protein dissociation and β-arrestin recruitment, thereby avoiding signal amplification [12].

As a chemokine receptor, CCR7 mainly couples to the Gαi/o subfamily of G proteins. Stimulation with CCL19 and CCL21 indeed revealed the activation of all members of the cognate Gαi/o family across the different cellular backgrounds tested, with similar potency and efficacy. Some studies that described differences between both ligands with regard to G protein activation looked at the inhibition of cAMP accumulation or probed G protein recruitment to the receptor but not the direct dissociation or conformational reorganisation of the heterotrimeric G proteins following receptor activation [23,26,27]. Interestingly, another study that investigated direct G protein activation through GTPγs binding also showed similar activity for CCL19 and CCL21 [22]. As such, there might be a discrepancy between the recruitment of G proteins to the receptor and their subsequent activation and dissociation.

Non-cognate couplings were observed in a cell-type-dependent manner. For instance, in U87 and CHO-K1 cells, Gαq was activated, but it was not activated in HEK293A cells. Some G proteins were uniquely activated in response to one of the two ligands. For instance, in CHO-K1 cells, Gα12 was only activated by CCL21 and GαsL was only activated by CCL19. β-arrestins were also recruited across all backgrounds through both CCL19 and CCL21 stimulation. However, their potency, but not efficacy, was strongly cell-type-dependent. This is in line with other studies, which report CCL21 to be significantly worse at inducing β-arrestin2 recruitment [21,22,23,26,27]. In a previous study, we obtained similar results regarding G protein activation and β-arrestin recruitment [24]. Here, we used NanoBRET instead of NanoBiT to monitor β-arrestin recruitment. Both techniques resulted in a significant potency difference with regard to β-arrestin2 recruitment, but the fold difference using NanoBRET was markedly smaller. This shows that the employed method can have an effect on the results even when the same equipment and cell lines are used.

Quantifying ligand bias is not a trivial task, and new methods are being developed with staggering speed. One prevalent method is fitting the operational model of agonism directly to the data [11,12]. However, fitting this model is cumbersome and requires additional information such as binding data. Recently, a new method was proposed that determines a ‘functional K_A_’ from fitting dose-responses on all acquired data simultaneously, circumventing the need for binding data [32]. Here, we opted for a method using an approximation of the transducer coefficient based on potencies and efficacies, which was previously proposed as a standardised method for ligand bias quantification [12]. The calculated Log(E_max_/EC50) is similar to the transduction coefficient, Log(*τ*/K_A_), acquired from fitting the operational model of agonism when the Hill Slope is close to unity. Proper assessment of ligand bias requires testing in the same cellular system since receptor, transducer and effector stoichiometry can influence signalling [11,12,13]. By quantifying ligand bias relative to a reference ligand, we can eliminate the effects of this system bias. CCL19- and CCL21-mediated G protein activation patterns were fairly well conserved. Consequently, in the same cellular system, we found little to no ligand bias between cognate G proteins. A notable exception was a slight bias away from Gαi2 in CHO-K1 cells. With regard to β-arrestin, a weak bias was observed towards most G proteins in U87 cells.

Observed biases were not consistent between the different cellular systems, as indicated by the Gαi2 bias observed in CHO-K1 cells, but not other cell types. In our overexpression system, the variation in Gα expression within a cell system was minimal, as reflected by the basal RLU values prior to stimulation (Supplementary Figure S3A). However, U87 cells showed a consistently lower Gα expression as they are harder to transfect than HEK293A and CHO-K1 cells (Supplementary Figure S3B). Furthermore, all cell lines had similar stable CCR7 expression levels (Supplementary Figure S1). As G proteins are the first link in the downstream signalling cascade, their consistent expression should limit system variations, so other factors such as different post-translational modifications might be at play. In contrast to G proteins, β-arrestin recruitment is preceded by the GRK-mediated phosphorylation of the intracellular part of the receptor. Which GRK that phosphorylates activated CCR7 has previously been shown to be ligand-dependent, meaning that CCR7 stimulation by CCL19 or CCL21 leads to differential GRK–receptor interactions, which might imply a ligand bias [21]. In this case, changes in the expression level and ratios of GRKs in the different cell systems could explain these observed differences. The recent literature states that biased signalling is not necessarily conserved across cell types, highlighting the importance of characterising the cellular system used and the need for physiologically relevant assays [13]. Indeed, it was shown that μ-opioid receptor ligands endomorphin-1 and endomorhping-2 were biased toward β-arrestin2 recruitment over G protein activation in HEK293 cells, but only endomorhpin-1 displayed biased signalling in CHO-K1 cells [13]. In other words, if CCL19 and CCL21 differentially recruit GKRs, then the expression of these transducers could influence the observed β-arrestin recruitment bias.

In our study we employed three commonly used epithelial cell lines, one being of non-human origin. It warrants further investigation to see if the effect of the cellular system might be further amplified when ligand bias is studied in primary cells that endogenously express CCR7, such as mature dendritic cells or naïve T cells and B cells.

Interestingly, we previously reported that CCL19- and CCL21-induced distinct cellular impedance profiles and correlated this with differences in β-arrestin2 recruitment in HEK293A cells [24]. It seems, therefore, that even though no strong ligand bias was found in HEK293A cells, subtle differences in pathway activation can still manifest a difference in more physiologically relevant readouts. The methods for quantifying ligand bias outlined above have proven their value when screening novel compounds with maximal ligand bias compared to a well-established balanced reference agonist [33]. While quantifying ligand bias between endogenous agonists is certainly possible, the interpretation of the results is much more complicated. This work further supports the notion that ligand bias is not the only aspect of biased signalling that should be considered.

In conclusion, the presented study shows that the endogenous CCR7 ligands, CCL19 and CCL21, show largely conserved pathway preferences, which may slightly differ depending on the cellular background. Only weak, non-significant ligand bias was detected, mainly with β-arrestin in U87 cells and Gαi2 in CHO-K1 cells. Together, these data suggest that CCL19 and CCL21 share mostly overlapping, weakly biased transducer profiles, which can be influenced by the cellular context. How, and if, these smaller biases are relevant in a physiological context remains to be elucidated.

## Figures and Tables

**Table 1:** An overview of potency (pEC50), efficacy (E_max_; % of max CCL19 activity) and Log(E_max_/EC50) of CCL19 and CCL21 for transducers in multiple cellular backgrounds.

Transducer	Ligand	HEK293A			U87			CHO-K1		
		pEC50	E_max_	Log(E_max_/EC50)	pEC50	E_max_	Log(E_max_/EC50)	pEC50	E_max_	Log(E_max_/EC50)
Gαi1	CCL19	8.63 ± 0.06	94.69 ± 1.39	8.61 ± 0.06	8.28 ± 0.34	95.77 ± 1.30	8.26 ± 0.34	8.54 ± 0.09	96.06 ± 2.50 *	8.82 ± 0.09
	CCL21	8.47 ± 0.38	89.00 ± 7.21	8.41 ± 0.41	8.02 ± 0.54	93.08 ± 4.36	7.98 ± 0.55	8.37 ± 0.34	89.50 ± 14.03	8.32 ± 0.35
Gαi2	CCL19	9.03 ± 0.15	94.99 ± 4.25	9.01 ± 0.15	8.56 ± 0.09	94.22 ± 1.06	8.53 ± 0.08	8.69 ± 0.39	83.61 ± 6.65	8.61 ± 0.38
	CCL21	8.76 ± 0.24	95.68 ± 2.29	8.74 ± 0.24	8.27 ± 0.20	80.01 ± 16.70	8.16 ± 0.29	8.91 ± 0.40	103.51 ± 14.10	8.93 ± 0.41
Gαi3	CCL19	9.61 ± 0.16	96.94 ± 1.21	9.60 ± 0.16	8.87 ± 0.21	94.05 ± 5.35	8.85 ± 0.20	8.84 ± 0.19	88.66 ± 2.67	8.78 ± 0.20
	CCL21	9.33 ± 0.05	91.97 ± 3.19	9.30 ± 0.04	8.60 ± 0.26	90.76 ± 2.47	8.55 ± 0.26	8.84 ± 0.24	86.61 ± 20.68	8.77 ± 0.34
GαoA	CCL19	8.30 ± 0.12 *	96.18 ± 3.38	8.28 ± 0.13 *	8.59 ± 0.21	94.63 ± 5.54	8.57 ± 0.21	9.37 ± 0.38	93.04 ± 4.17	9.34 ± 0.37
	CCL21	7.97 ± 0.08	89.12 ± 1.59	7.92 ± 0.08	8.33 ± 0.37	96.17 ± 23.05	8.30 ± 0.84	9.27 ± 0.26	90.49 ± 9.19	9.22 ± 0.28
GαoB	CCL19	8.85 ± 0.21	98.46 ± 0.84	8.85 ± 0.21	8.80 ± 0.06	97.07 ± 2.33	8.78 ± 0.05	8.96 ± 0.22	93.86 ± 3.45	8.93 ± 0.23
	CCL21	8.34 ± 0.27	92.10 ± 9.87	8.30 ± 0.32	8.45 ± 0.19	101.87 ± 0.83	8.45 ± 0.19	8.97 ± 0.46	81.95 ± 17.33	8.87 ± 0.46
Gαq	CCL19	n.d.			8.41 ± 0.87	72.23 ± 26.73	8.24 ± 0.68	8.77 ± 0.63	78.38 ± 15.20	8.65 ± 0.65
	CCL21	n.d.			8.10 ± 0.65	78.50 ± 59.03	7.80 ± 0.47	8.45 ± 0.66	53.05 ± 22.37	8.15 ± 0.77
Gα12	CCL19	7.44 ± 0.26 *	91.44 ± 8.48 *	7.40 ± 0.25	7.81 ± 0.21	91.27 ± 7.16	7.77 ± 0.18	n.d.		
	CCL21	6.79 ± 0.20	149.83 ± 22.74	6.97 ± 0.13	7.50 ± 0.46	88.78 ± 21.52	7.44 ± 0.40	7.94 ± 0.11	70.30 ± 29.18	7.77 ± 0.07
Gα13	CCL19	n.d.			8.77 ± 0.23	84.19 ± 17.93	8.69 ± 0.33	8.87 ± 0.67	87.66 ± 6.56	8.81 ± 0.68
	CCL21	n.d.			8.70 ± 0.6	104.69 ± 31.37	8.71 ± 0.67	8.53 ± 0.73	77.97 ± 22.39	8.40 ± 0.68
Gα15	CCL19	7.80 ± 0.26 **	97.07 ± 5.41	7.78 ± 0.23 *	n.d.			8.91 ± 1.02	78.62 ± 10.63	8.80 ± 0.96
	CCL21	6.74 ± 0.22	164.09 ± 57.96	6.94 ± 0.11	n.d.			7.56 ± 0.31	87.78 ± 15.35	7.50 ± 0.29
GαsS	CCL19	n.d.			8.65 ± 0.41	89.30 ± 4.17	8.60 ± 0.39	8.58 ± 0.29 *	83.38 ± 8.74	8.50 ± 0.28 *
	CCL21	231.28 ± 174.49	7.13 ± 0.83	7.42 ± 0.53	8.39 ± 0.49	104.80 ± 29.32	8.40 ± 0.50	8.10 ± 0.15	76.48 ± 16.16	7.98 ± 0.19
GαsL	CCL19	7.49 ± 0.54	88.20 ± 20.66	7.43 ± 0.45	8.95 ± 1.33	82.58 ± 11.20	8.86 ± 1.27	8.97 ± 0.74	72.43 ± 7.06	8.82 ± 0.76
	CCL21	6.79 ± 0.73	170.51 ± 117.5	6.94 ± 0.44	8.99 ± 1.32	101.11 ± 16.89	8.99 ± 1.31	n.d.		
Arrβ1	CCL19	7.46 ± 0.14	108.79 ± 3.73	7.49 ± 0.13	7.86 ± 0.24 **	104.49 ± 3.69	7.88 ± 0.23 **	7.92 ± 0.25	105.06 ± 2.93	7.94 ± 0.24
	CCL21	7.15 ± 0.17	107.66 ± 3.12	7.19 ± 0.17	6.90 ± 0.15	106.43 ± 5.12	6.92 ± 0.13	7.52 ± 0.25	102.60 ± 4.96	7.53 ± 0.23
Arrβ2	CCL19	7.57 ± 0.13 *	106.42 ± 5.36	7.59 ± 0.11 **	7.86 ± 0.16 **	102.06 ± 4.66	7.86 ± 0.15 **	8.11 ± 0.29	102.10 ± 5.79	8.12 ± 0.27
	CCL21	7.02 ± 0.05	106.86 ± 1.87	7.05 ± 0.05	7.01 ± 0.15	97.20 ± 5.27	7.00 ± 0.16	7.69 ± 0.27	98.30 ± 4.19	7.68 ± 0.28

Data represent the mean and SD of three to six independent experiments. SD values of Log(E_max_/EC50) were calculated through the standard propagation of error. Differences between CCL19 and CCL21 for each feature were analysed using an unpaired *t*-test with Welch’s correction. Values in bold indicate statistical significance where * and ** represent *p* < 0.05 and 0.01, respectively. n.d. values could not be determined due to a lack of activation.

## Data Availability

Data are contained within the article or Supplementary Materials.

## Supplementary Materials

The following supporting information can be downloaded via this link: https://www.mdpi.com/article/10.3390/bios14030142/s1, Figure S1: CCR7 expression in different cell lines; Figure S2: Transducer activation and recruitment by CCR7; Figure S3: G protein expression levels between transducers and cell lines; Table S1: Overview of ligand preference at CCR7; Table S2: Overview of system preference at CCR7.
