# Supplementary material for: Systematic Assessment of Human CCR7 Signalling Using NanoBRET Biosensors Points towards the Importance of the Cellular Context
Source: Biosensors (Basel). 2024 Mar 14;14(3):142. doi: 10.3390/bios14030142 (PMC10967792; doi:10.3390/bios14030142)
Supplement: Supplementary file 1 [file biosensors-14-00142-s001.zip › biosensors-2895665-supplementary.pdf]

Supplemental Table S1. Overview of ligand preference at CCR7. Ligand preference ($\Delta Log(E_{max}/EC50)$) with CCL19 as a reference was calculated by subtracting $Log(E_{max}/EC50)$ in each pathway. Data are representative of the mean and SD of three to six independent experiments. SD values were calculated through standard propagation of error. Differences between CCL19 and CCL21 were analysed using an unpaired t-test with Welch's correction. * and ** represent $P < 0.05$ and 0.01 respectively.

Cell type	Ligand	Gαi1	Gαi2	Gαi3	GαoA	GαoB	Gαq	Gα12	Gα13	Gα15	GαsS	GαsL	Arrβ1	Arrβ2
HEK293A	CCL19 (ref)	0.00 ± 0.08	0.00 ± 0.22	0.00 ± 0.23	0.00 ± 0.18	0.00 ± 0.30	n.d.	0.00 ± 0.35	n.d.	**0.00 ± 0.33 ***	n.d.	0.00 ± 0.63	0.00 ± 0.18	**0.00 ± 0.16 ***
	CCL21	-0.19 ± 0.41	-0.27 ± 0.28	-0.30 ± 0.17	-0.37 ± 0.15	-0.54 ± 0.38	n.d.	-0.43 ± 0.28	n.d.	**-0.84 ± 0.26 ***	n.d.	-0.49 ± 0.63	-0.31 ± 0.21	**-0.54 ± 0.12 ***
U87	CCL19 (ref)	0.00 ± 0.48	0.00 ± 0.12	0.00 ± 0.28	0.00 ± 0.29	0.00 ± 0.08	0.00 ± 0.96	0.00 ± 0.26	0.00 ± 0.47	n.d.	0.00 ± 0.55	0.00 ± 1.79	**0.00 ± 0.33 ***	**0.00 ± 0.21 ****
	CCL21	-0.28 ± 0.65	-0.37 ± 0.30	-0.29 ± 0.33	-0.27 ± 0.52	-0.33 ± 0.20	-0.44 ± 0.83	-0.33 ± 0.44	0.02 ± 0.75	n.d.	-0.20 ± 0.63	0.13 ± 1.82	**-0.96 ± 0.27 ***	**-0.87 ± 0.21 ****
CHO-K1	CCL19 (ref)	0.00 ± 0.13	0.00 ± 0.54	0.00 ± 0.28	0.00 ± 0.52	0.00 ± 0.32	0.00 ± 0.92	n.d.	0.00 ± 0.97	0.00 ± 1.35	0.00 ± 0.40	0.00 ± 1.08	0.00 ± 0.34	0.00 ± 0.38
	CCL21	-0.21 ± 0.36	0.31 ± 0.56	-0.01 ± 0.39	-0.11 ± 0.46	-0.06 ± 0.51	-0.51 ± 1.01	n.d.	-0.40 ± 0.97	-1.30 ± 1.00	-0.52 ± 0.34	n.d.	-0.41 ± 0.33	-0.44 ± 0.39

Supplemental Table S2. Overview of system preference at CCR7. System preference (ΔLog(E_{max}/EC50)) were calculated for each ligand by subtracting the Log(E_{max}/EC50) value of a reference system from all other systems for each transducer and each ligand (Eq. 5). Data are representative of the mean and SD of three to six independent experiments. SD values were calculated through standard propagation of error. A one-way ANOVA followed by a Dunnett multiple comparison was used to assess the preference of a system to the reference. * and ** represent P < 0.05 and 0.01 respectively.

Transducer	Cell type	HEK293A (ref)		U87 (ref)		CHO-K1 (ref)	
		CCL19	CCL21	CCL19	CCL21	CCL19	CCL21
Gαi1	HEK293A	0.00 ± 0.08	0.00 ± 0.58	0.35 ± 0.35	0.43 ± 0.68	0.09 ± 0.11	0.10 ± 0.54
	U87	-0.35 ± 0.35	-0.43 ± 0.68	0.00 ± 0.48	0.00 ± 0.77	-0.26 ± 0.35	-0.33 ± 0.65
	CHO-K1	-0.09 ± 0.11	-0.10 ± 0.54	0.26 ± 0.35	0.33 ± 0.65	0.00 ± 0.13	0.00 ± 0.49
Gαi2	HEK293A	0.00 ± 0.22	0.00 ± 0.33	0.48 ± 0.18	0.58 ± 0.37	0.40 ± 0.41	-0.18 ± 0.47
	U87	-0.48 ± 0.18	-0.58 ± 0.37	0.00 ± 0.12	0.00 ± 0.41	-0.08 ± 0.39	-0.76 ± 0.50
	CHO-K1	-0.40 ± 0.41	0.18 ± 0.47	0.08 ± 0.39	0.76 ± 0.50	0.00 ± 0.54	0.00 ± 0.57
Gαi3	HEK293A	0.00 ± 0.23	0.00 ± 0.06	**0.75 ± 0.26** *	0.74 ± 0.26	**0.81 ± 0.26** *	0.52 ± 0.34
	U87	**-0.75 ± 0.26** *	**-0.74 ± 0.26** *	0.00 ± 0.28	0.00 ± 0.37	0.06 ± 0.28	-0.22 ± 0.43
	CHO-K1	**-0.81 ± 0.26** *	-0.52 ± 0.34	-0.06 ± 0.28	0.22 ± 0.43	0.00 ± 0.28	0.00 ± 0.48
GαoA	HEK293A	0.00 ± 0.18	0.00 ± 0.12	-0.28 ± 0.25	-0.39 ± 0.48	**-1.05 ± 0.39** *	**-1.31 ± 0.29** **
	U87	0.28 ± 0.25	0.39 ± 0.48	0.00 ± 0.29	0.00 ± 0.67	-0.77 ± 0.42	**-0.92 ± 0.55** *
	CHO-K1	**1.05 ± 0.39** **	**1.31 ± 0.29** **	0.77 ± 0.42	0.92 ± 0.55	0.00 ± 0.52	0.00 ± 0.39
GαoB	HEK293A	0.00 ± 0.30	0.00 ± 0.45	0.06 ± 0.22	-0.15 ± 0.37	-0.08 ± 0.31	-0.57 ± 0.56
	U87	-0.06 ± 0.22	0.15 ± 0.37	0.00 ± 0.08	0.00 ± 0.27	-0.15 ± 0.23	-0.42 ± 0.50
	CHO-K1	0.08 ± 0.31	0.57 ± 0.56	0.15 ± 0.23	0.42 ± 0.50	0.00 ± 0.32	0.00 ± 0.65
Gαq	HEK293A	n.d.	n.d.	n.d.	n.d.	n.d.	n.d.
	U87	n.d.	n.d.	0.00 ± 0.96	0.00 ± 0.66	-0.42 ± 0.94	-0.35 ± 0.90
	CHO-K1	n.d.	n.d.	0.42 ± 0.94	0.35 ± 0.90	0.00 ± 0.92	0.00 ± 1.09
Gα12	HEK293A	0.00 ± 0.35	0.00 ± 0.19	-0.37 ± 0.31	-0.47 ± 0.42	n.d.	**-0.80 ± 0.15** *
	U87	0.37 ± 0.31	0.47 ± 0.42	0.00 ± 0.26	0.00 ± 0.57	n.d.	-0.33 ± 0.41
	CHO-K1	n.d.	**0.80 ± 0.15** *	n.d.	0.33 ± 0.41	n.d.	0.00 ± 0.10
Gα13	HEK293A	n.d.	n.d.	n.d.	n.d.	n.d.	n.d.
	U87	n.d.	n.d.	0.00 ± 0.47	0.00 ± 0.95	-0.12 ± 0.76	0.31 ± 0.96
	CHO-K1	n.d.	n.d.	0.12 ± 0.76	-0.31 ± 0.96	0.00 ± 0.97	0.00 ± 0.97
Gα15	HEK293A	0.00 ± 0.33	0.00 ± 0.15	n.d.	n.d.	-1.02 ± 0.99	-0.56 ± 0.31
	U87	n.d.	n.d.	n.d.	n.d.	n.d.	n.d.
	CHO-K1	1.02 ± 0.99	**0.56 ± 0.31** *	n.d.	n.d.	0.00 ± 1.35	0.00 ± 0.41
GαsS	HEK293A	n.d.	0.00 ± 0.75	n.d.	-0.98 ± 0.73	n.d.	-0.56 ± 0.56
	U87	n.d.	0.98 ± 0.73	0.00 ± 0.55	0.00 ± 0.70	0.10 ± 0.48	0.42 ± 0.53
	CHO-K1	n.d.	0.56 ± 0.56	-0.10 ± 0.48	-0.42 ± 0.53	0.00 ± 0.40	0.00 ± 0.27
GαsL	HEK293A	0.00 ± 0.63	0.00 ± 0.63	-1.43 ± 1.34	-2.05 ± 1.38	-1.39 ± 0.88	n.d.
	U87	1.43 ± 1.34	2.05 ± 1.38	0.00 ± 1.79	0.00 ± 1.85	0.04 ± 1.48	n.d.
	CHO-K1	1.39 ± 0.88	n.d.	-0.04 ± 1.48	n.d.	0.00 ± 1.08	n.d.
Arrβ1	HEK293A	0.00 ± 0.18	0.00 ± 0.24	-0.39 ± 0.26	0.26 ± 0.22	-0.45 ± 0.27	-0.34 ± 0.28
	U87	0.39 ± 0.26	-0.26 ± 0.22	0.00 ± 0.33	0.00 ± 0.19	-0.06 ± 0.33	-0.61 ± 0.26
	CHO-K1	0.45 ± 0.27	0.34 ± 0.28	0.06 ± 0.33	**0.61 ± 0.26** *	0.00 ± 0.34	0.00 ± 0.32
Arrβ2	HEK293A	0.00 ± 0.16	0.00 ± 0.07	-0.27 ± 0.18	0.06 ± 0.16	-0.53 ± 0.29	-0.63 ± 0.29
	U87	0.27 ± 0.18	-0.06 ± 0.16	0.00 ± 0.21	0.00 ± 0.22	-0.25 ± 0.31	-0.69 ± 0.32
	CHO-K1	**0.53 ± 0.29** *	**0.63 ± 0.29** *	0.25 ± 0.31	**0.69 ± 0.32** *	0.00 ± 0.38	0.00 ± 0.40

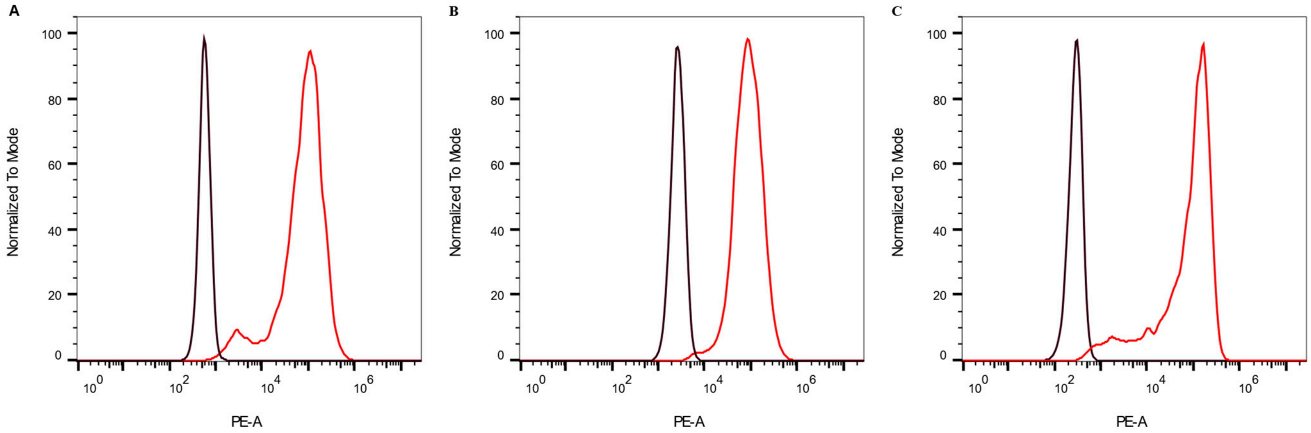

Supplementary Figure S1. CCR7 expression in different cell lines. (A) HEK293A, **(B)** U87, and **(C)** CHO-K1 cells stably expressing CCR7 were stained with PE Mouse anti-Human CCR7 (red) or PE Mouse IgG2a κ Isotype Control (black) and receptor expression was quantified using flow cytometry.

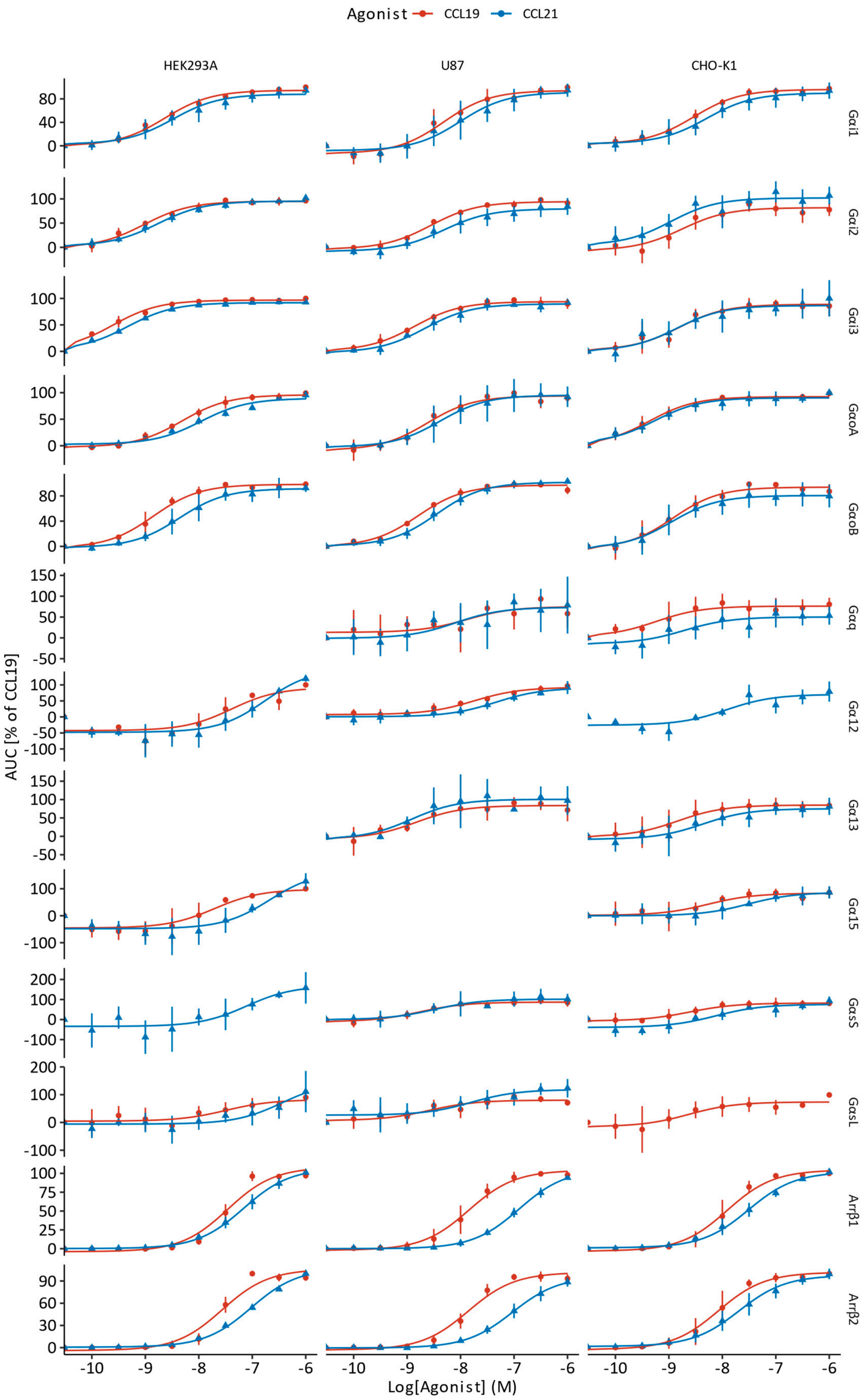

Supplementary Figure S2. Transducer activation and recruitment by CCR7. G protein dissociation and β-arrestin recruitment was measured in response to CCL19 (red) and CCL21 (blue) in HEK293A, U87 and CHO-K1 cells. Data are represented as the mean and SD of three to six independent experiments, each with three technical replicates. Curves were fit to a four-parameter non-linear regression model with the slope fixed to -1. Only data which showed significant activation or recruitment as determined by one-way ANOVA are shown.

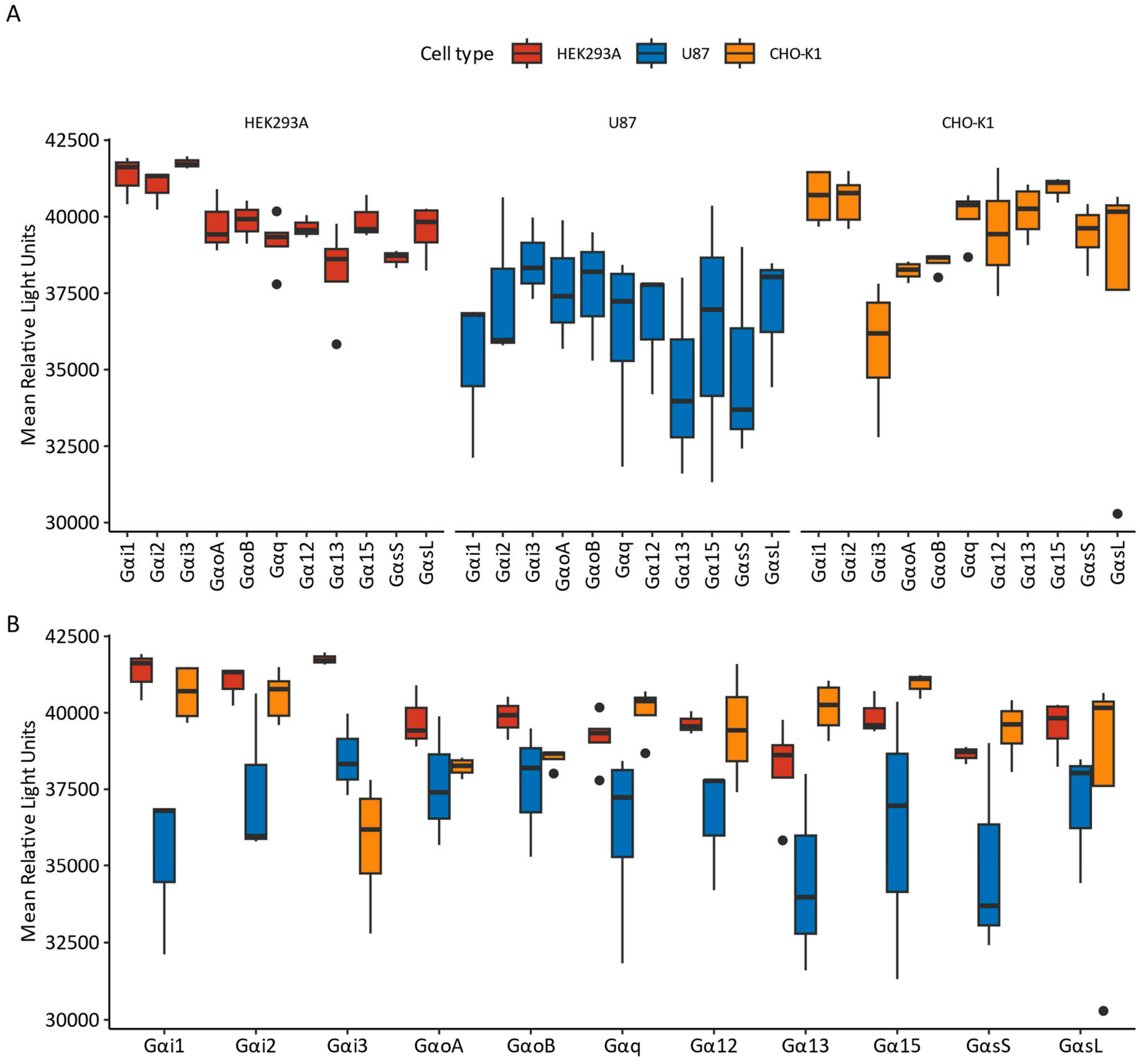

Supplementary Figure S3. G protein expression levels between transducers and cell lines. Comparison between mean relative light units (RLU) prior to ligand stimulation as a proxy for G protein expression between **(A)** G proteins within a single cell type or **(B) a** G protein across cell types. **(A-B)** Data are represented as the mean and SD of three to six independent experiments.
